# Epidemiology of ventilator-associated pneumonia in ICU COVID-19 patients: an alarming high rate of multidrug-resistant bacteria

**DOI:** 10.1186/s44158-022-00065-4

**Published:** 2022-08-19

**Authors:** Nardi Tetaj, Alessandro Capone, Giulia Valeria Stazi, Maria Cristina Marini, Gabriele Garotto, Donatella Busso, Silvana Scarcia, Ilaria Caravella, Manuela Macchione, Giada De Angelis, Rachele Di Lorenzo, Alessandro Carucci, Maria Vittoria Antonica, Ilaria Gaviano, Carlo Inversi, Elisabetta Agostini, Flaminia Canichella, Giorgia Taloni, Francesca Evangelista, Ilaria Onnis, Giulia Mogavero, Maria Elena Lamanna, Dorotea Rubino, Mattia Di Frischia, Candido Porcelli, Elena Cesi, Andrea Antinori, Fabrizio Palmieri, Gianpiero D’Offizi, Fabrizio Taglietti, Carla Nisii, Maria Adriana Cataldo, Stefania Ianniello, Paolo Campioni, Francesco Vaia, Emanuele Nicastri, Enrico Girardi, Luisa Marchioni, ICU COVID-19 Study Group, Adele Grisaro, Anna Farina, Ricardo Jose Cabas Merino, Sibiana Micarelli, Valeria Petroselli, Giuseppina Ragosta, Sara Zito

**Affiliations:** 1 National Institute for Infectious Diseases IRCCS Lazzaro Spallanzani, Rome, Italy; 2 UOC Resuscitation, Intensive and Sub-Intensive Care, National Institute for Infectious Diseases IRCCS Lazzaro Spallanzani, 00149 Rome, Italy; 3 Clinical and Research Department of Infectious Diseases, National Institute for Infectious Diseases IRCCS Lazzaro Spallanzani, 00149 Rome, Italy; 4 Health Direction, National Institute for Infectious Diseases IRCCS Lazzaro Spallanzani, 00149 Rome, Italy; 5 Scientific Direction, National Institute for Infectious Diseases IRCCS Lazzaro Spallanzani, 00149 Rome, Italy

**Keywords:** Ventilator-associated pneumonia, Intensive care unit, Coronavirus disease 2019, COVID-19, Acute respiratory distress syndrome, ARDS, Corticosteroid

## Abstract

**Background:**

COVID‑19 is a novel cause of acute respiratory distress syndrome (ARDS) that leads patients to intensive care unit (ICU) admission requiring invasive ventilation, who consequently are at risk of developing of ventilator‑associated pneumonia (VAP). The aim of this study was to assess the incidence, antimicrobial resistance, risk factors, and outcome of VAP in ICU COVID-19 patients in invasive mechanical ventilation (MV).

**Methods:**

Observational prospective study including adult ICU admissions between January 1, 2021, and June 31, 2021, with confirmed COVID-19 diagnosis were recorded daily, including demographics, medical history, ICU clinical data, etiology of VAPs, and the outcome. The diagnosis of VAP was based on multi-criteria decision analysis which included a combination of radiological, clinical, and microbiological criteria in ICU patients in MV for at least 48 h.

**Results:**

Two hundred eighty-four COVID-19 patients in MV were admitted in ICU. Ninety-four patients (33%) had VAP during the ICU stay, of which 85 had a single episode of VAP and 9 multiple episodes. The median time of onset of VAP from intubation were 8 days (IQR, 5–13). The overall incidence of VAP was of 13.48 episodes per 1000 days in MV. The main etiological agent was *Pseudomonas aeruginosa* (39.8% of all VAPs) followed by *Klebsiella spp*. (16.5%); of them, 41.4% and 17.6% were carbapenem resistant, respectively. Patients during the mechanical ventilation in orotracheal intubation (OTI) had a higher incidence than those in tracheostomy, 16.46 and 9.8 episodes per 1000-MV day, respectively. An increased risk of VAP was reported in patients receiving blood transfusion (OR 2.13, 95% CI 1.26–3.59, *p* = 0.005) or therapy with Tocilizumab/Sarilumab (OR 2.08, 95% CI 1.12–3.84, *p* = 0.02). The pronation and PaO_2_/FiO_2_ ratio at ICU admission were not significantly associated with the development of VAPs. Furthermore, VAP episodes did not increase the risk of death in ICU COVID-19 patients.

**Conclusions:**

COVID-19 patients have a higher incidence of VAP compared to the general ICU population, but it is similar to that of ICU ARDS patients in the pre-COVID-19 period. Interleukin-6 inhibitors and blood transfusions may increase the risk of VAP. The widespread use of empirical antibiotics in these patients should be avoided to reduce the selecting pressure on the growth of multidrug-resistant bacteria by implementing infection control measures and antimicrobial stewardship programs even before ICU admission.

**Supplementary Information:**

The online version contains supplementary material available at 10.1186/s44158-022-00065-4.

## Background

Coronavirus disease 2019 (COVID-19) is an infectious disease caused by a coronavirus discovered in December 2019, named severe acute respiratory syndrome corona-virus-2 (SARS-CoV-2), and it is responsible for the current global pandemic that started in March 2020 [1].

COVID-19 can cause lung complications such as pneumonia and in the most severe cases ARDS. In COVID-19 patients with persistent hypoxemia who are unresponsive to non-invasive ventilation (NIV), orotracheal intubation (OTI) is necessary and ICU admission is required in patients with persistent hypoxemia [2]. In the event of expected long-term mechanical ventilation or failed weaning, tracheostomy is indicated [3].

Ventilator-associated pneumonia (VAP), one of the most common health care-related infectious complications of invasive mechanical ventilation, may increase the intensity of medical care and also complicate the outcome of ICU patients.

Koulenti et al. in 2016 reported in the EU-VAP/CAP study, a multicenter retrospective observational study referring to a period from 1999 to 2006, enrolling patients from 27 ICUs in nine European countries, the incidence of VAP with 18.3 events per 1000 ventilator days [4]. In the past decade, there has been a reduction in the incidence of VAPs in European ICUs, probably due to better implementation of preventive strategies [5]. Indeed, more recently, in 2019, the European Centre for Disease Prevention and Control (ECDC) reported an incidence of VAP of 9.5 episodes per 1000 intubation days, based on data from 1192 hospitals and 1480 ICUs, where 14 countries participated [6].

Furthermore, the Italian PROSAFE project report of 2018, performed by GiViTi (Italian Group for the Evaluation of Interventions in Intensive Care) which includes data collected from 122 ICUs in Italy, found that the incidence of VAP in patients mechanically ventilated for at least 8 days was 9.8 events per 1000 ventilator days (CI 95%, 9.2–10.4) [7].

Forel et. al. in 2012 showed that VAP occurred in almost one third of patients with acute respiratory distress syndrome (ARDS) in intensive care unit (ICU) and were associated with an increase ICU mortality which did not remain significant after adjustment [8].

The aforementioned studies refer to the pre-COVID-19 era. Data in literature show that patients with COVID-19 may have a higher risk of developing VAP than patients without COVID-19, as reported in two recent multicenter Italian studies performed in 2021 on COVID-19 patients by Grasselli et al. and Giacobbe et al., with an incidence of 18 and 26 events per 1000 ventilator days [9, 10], respectively. A further study conducted in France by Blonz et al. reports an incidence of VAPs of 39.0 episodes per 1000 ventilator days [11]. Few of these studies have investigated risk factors. Still, incidence, risk factors, and mortality rates associated with VAP in ARDS COVID-19 patients are unclear and a matter of debate [11, 12].

The primary objective of this prospective observational study was to assess the prevalence and incidence rates of VAP in COVID-19 patients in ICU. The secondary objective was to assess the frequency of microbial isolates, pattern of antimicrobial resistance, risk factors associated with VAP, and ICU mortality in VAP patients.

## Material and methods

### Study design and definitions

A prospective cohort study was conducted at the “Lazzaro Spallanzani” National Institute for Infectious Disease (INMI) in Rome, Italy, which is a third level COVID-19 center with 200-bed hospital for infectious disease and 55-bed in ICU.

In the present study, adult COVID-19 patients admitted to our ICU from January 1, 2021, to June 31, 2021, and underwent invasive mechanical ventilation for at least 7 days were included.

Patients transferred to another hospital and those still hospitalized at ICU at the end of the study were excluded. COVID-19 was diagnosed by nasal pharyngeal swab for reverse transcriptase polymerase chain reaction (rt-PCR) assay. The diagnosis of VAP was based on multi-criteria decision analysis adapted from the ECDC recommendations [5] and Clinical Practice Guidelines by the Infectious Diseases Society of America and the American Thoracic Society [13], which included a combination of radiological, clinical, and microbiological criteria in ICU patients in invasive mechanical ventilation (MV) for at least 48 h. VAPs were diagnosed by Clinical Research Coordinators based on their experience in clinical practice and publications. They also contributed to the supervision and the review of the study.

The clinical criteria included at least one of the following signs: fever > 38 °C with no other cause, leukocytes < 4000/mm^3^ or > 12,000/mm^3^, and at least one of the following: new onset of purulent sputum or change in character of sputum (color, odor, quantity, consistency), worsening gas exchange – PaO_2_/FiO_2_ ratio which is the ratio of arterial partial pressure (PaO_2_) to fractional inspired oxygen (FiO_2_). The radiological criteria included new or worsening infiltrates on lung CT. Regarding microbiological criteria the American guidelines [13] recommend non-invasive and semi-quantitative sampling, and European guidelines (ECDC) [5] recommend invasive and quantitative sampling. In our study, serial blind bronchoalveolar lavages were performed every 72 h in every intubated patient with the semi-quantitative sampling. In case of rare species, which corresponds from 10^3^ to 10^4^ CFU/ml in our microbiology laboratory in semi-quantitative sampling, the etiologic agent has not been considered and the VAP was not diagnosed. Positive quantitative culture from minimally contaminated lower respiratory tract (LRT) specimen was considered a threshold of 10^4^ colony-forming units (CFU)/mL for bronchoscopy bronchoalveolar lavage; or a threshold of 10^5^ CFU/ml from endotracheal aspirate.

Patients on MV up to weaning were given continuous intravenous sedatives to help attenuate the anxiety, pain, and agitation associated with mechanical ventilation. Nutritional support in ICU was performed according to the European Society for Clinical Nutrition and Metabolism (ESPEN) guidelines [14].

Percutaneous tracheostomy was performed in patients with prolonged mechanical ventilation and weaning failure from mechanical ventilator [15].

### Data collection

For each enrolled patient, the following data were prospectively collected: age, gender, and body mass index (BMI); sequential organ failure assessment score (SOFA score), acute physiology and chronic health evaluation II score (APACHE II score), and PaO_2_/FiO_2_ at ICU admission; pre-ICU hospitalization as days of hospitalization in the ward until admission to intensive care, ICU length of stay, and etiology of VAPs. Also, corticosteroid therapy, antiviral therapy (tocilizumab or sarilumab), previous antibiotic therapy (30 days before orotracheal intubation), blood transfusions during ICU stay, hemodialysis, barotrauma (pneumothorax or pneumomediastinum), and pronation at least 1 cycle of 12 h were collected during the ICU hospitalization.

The comorbidities included arterial hypertension, cardiovascular diseases, diabetes, obesity (defined as BMI > 30 kg/m^2^), chronic renal disease in stages 3–5 of CKD (chronic kidney disease), chronic liver disease (moderate to severe liver disease), chronic lung diseases (which included chronic obstructive pulmonary disease—COPD, bronchial asthma, emphysema, or other pulmonary diseases), previous neoplasm (during the last 5 years, which includes solid neoplasia or haematological malignancy), chronic neurological disorders, autoimmune diseases, and other chronic diseases (which includes all other less common diseases). We also recorded previous hospitalization in last 6 months, previous surgery in the last month. During the ICU hospitalization of included patients, Clinical Research Coordinators as trained healthcare personnel assessed patients for the occurrence of VAP. Finally, a 30-day and 60-day-mortality were recorded, along with the total length of ICU hospitalization and of IVM, performing of tracheostomy.

### Statistical analysis

The prevalence rate of VAP was calculated by dividing the number of patients who acquired VAP by the total number of included patients. The incidence rate of VAP was calculated as number of episodes/1000 days of invasive mechanical ventilation in both orotracheal intubation and tracheostomy procedures.

To assess potential risk factors associated with VAP, patients with VAP were compared with patients who did not acquire VAP during ICU stay.

Quantitative variables are expressed as medians (interquartile range, IQR), while categorical variables were expressed as counts (N) and percentages (%). The statistical comparison was performed by means of the Mann–Whitney test for continuous variables and chi-square test (or Fisher or chi-square test for trend where necessary) for categorical variables.

Potential risk factors for VAP were analysed by univariate analysis. Two-tailed *p* values < 0.05 were considered statistically significant. Variables from the univariate analysis were considered for inclusion in multivariate logistic regression analysis if *p* value was less than 0.05. Backward stepwise logistic regression was performed. Survival curves of patients with and without VAP were obtained using the Kaplan–Meier method. The statistical analysis was performed SPSS Statistics 27 (IBM Corp, Armonk, New York, USA).

## Results

During the study period, 451 COVID-19 patients were hospitalized in our intensive care unit; 121 (26.8%) of them were treated with noninvasive ventilation in our ICU and 330 (73.2%) patients underwent OTI, and of them, 133 patients (40.3%) were tracheostomized.

**Fig. 1:**
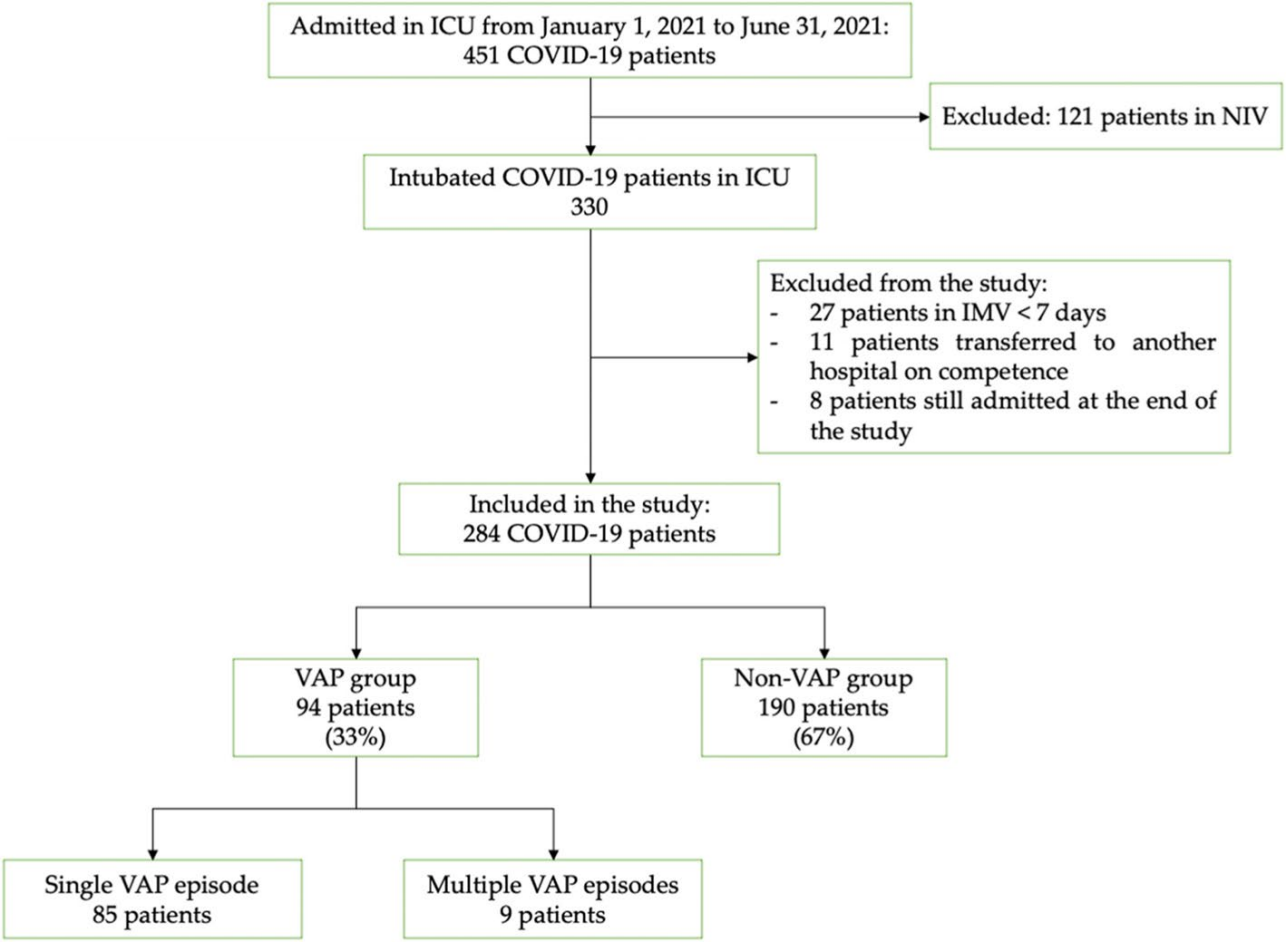
Flowchart of study selection. COVID-19, coronavirus 2019; ICU, intensive care unit; IMV, invasive mechanical ventilation; NIV, non-invasive ventilation; VAP, ventilator-associated pneumonia

We excluded from the study 46 patients: 27 patients underwent invasive mechanical ventilation for less than 7 days; 10 patients were still admitted at the end of the study; 11 patients were transferred to other hospitals for competence, of which 6 patients transferred to an extracorporeal membrane oxygenation center (ECMO), 3 patients to a coronary care unit, and 2 patients to a surgical care unit (Fig. 1).

### Baseline characteristics of COVID-19 patients with VAP

**Fig. 2:**
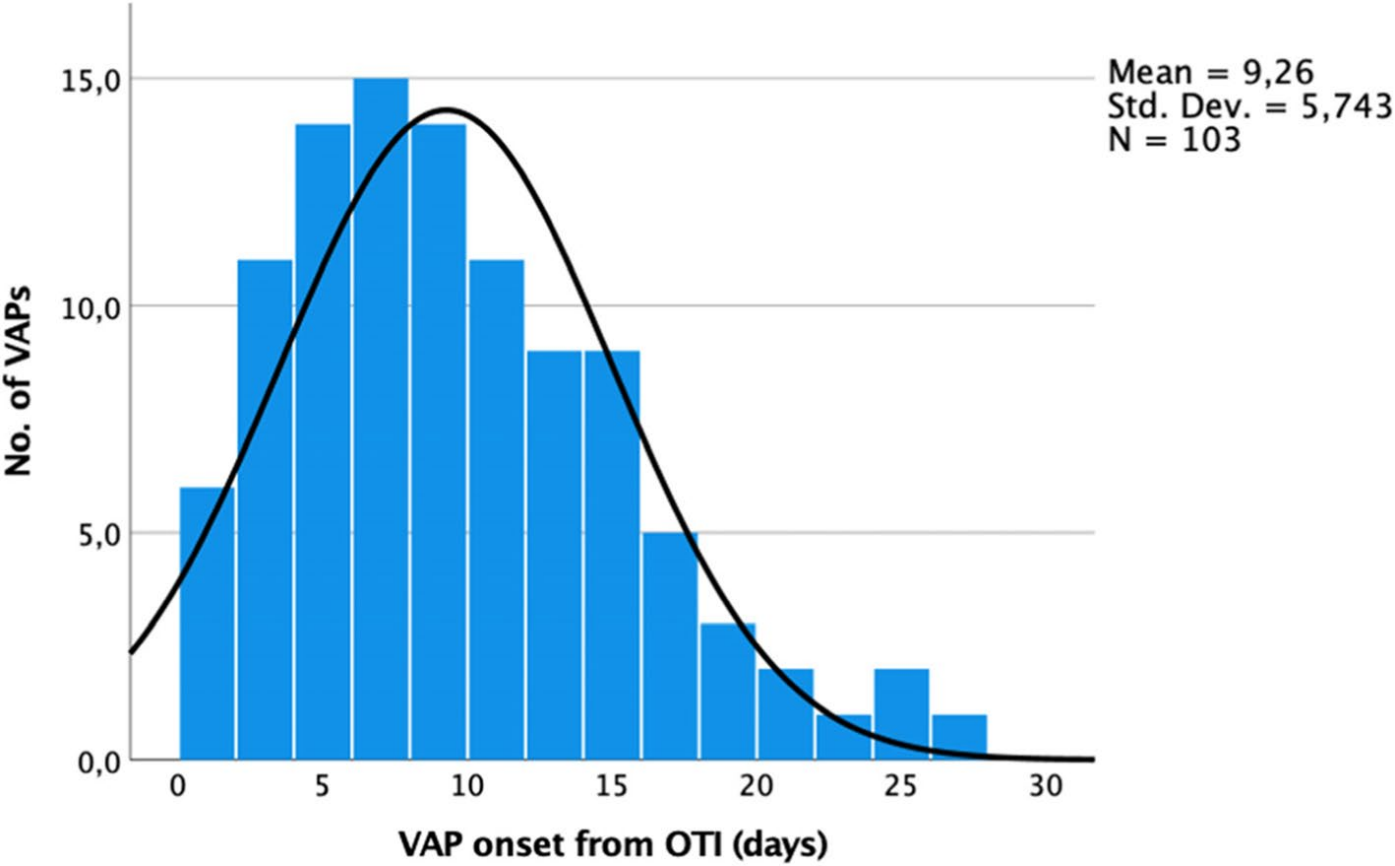
Timeline of onset of VAP from OTI. VAP, ventilator-associated pneumonia; OTI, orotracheal intubation

Demographics, comorbidities, and clinical features of ICU COVID-19 patients in MV with and without VAP are summarized in Table 1. The median age was 66 years (IQR, 58–73), and 71.5% were male. The median BMI was 27.7 kg/m^2^ (IQR, 25–31), and median SOFA score and APACHE score at ICU admission were respectively 4.5 (IQR, 3–7) and 12 (IQR, 8–17). The most frequent comorbidities were arterial hypertension (54.9%), obesity (35.2%), cardiovascular disease (23.2%), diabetes (17.6%), and chronic lung disease (13.4%). The mean pre-ICU hospitalisation was 6 days (SD, ± 8.0). The mean days on MV were 26.9 (SD, ± 19.9), with a total accumulative time of 7638 days on mechanical ventilation. The mean ICU length of stay was 29.3 days (SD, ± 19.8). The timeline distribuition of VAPs from OTI is shown on Fig. 2. The median time to onset of VAP from intubation were 8 days (IQR, 5–13). Twenty-one patients (22%) had early-onset VAP (within the first 4 days of MV) and 73 of them (78%) had late-onset VAP (> 4 days MV). A comparison of patient demographics, clinical features, and etiological between early and late VAP are shown in Tables 1 and 2 of the supplemental material.

**Table 1:** Baseline demographic and clinical characteristics of patients at ICU admission

Characteristics	Total	VAP	No VAP	*p* value ^a^
	284	94	190	
Age, median (IQR)	66 (58–73)	66.5 (57–73)	66 (58–74)	0.806
Male, ***n*** (%)	203 (71.5)	60 (63.8)	143 (75.3)	0.245
Female, ***n*** (%)	81 (28.5)	34 (36.2)	47 (24.7)	0.245
BMI, kg/m^**2**^, median (IQR)	27.7 (25–31)	28.2 (25.4–32.4)	27.6 (25–30.5)	0.134
SOFA score, median* (IQR)	4.5 (3–7)	6 (4–8)	4 (3–7)	**0.007**
APACHE II score, median* (IQR)	12 (8–17)	13 (9–19)	11.5 (7–16)	0.068
**Comorbidities, *n* (%)**				
Arterial hypertension	156 (54.9)	51 (54.3)	105 (55.2)	0.873
Cardiovascular diseases	66 (23.2)	28 (29.8)	38 (20.0)	0.067
Diabetes	50 (17.6)	17 (18.1)	33 (17.3)	0.897
Obesity ^a^	100 (35.2)	40 (42.5)	60 (31.6)	0.069
Chronic renal disease ^b^	13 (4.6)	5 (5.3)	8 (4.2)	0.675
Chronic liver disease	3 (1.0)	3 (1.6)	0 (0.0)	N.A
Chronic lung disease	38 (13.4)	16 (17.0)	22 (11.6)	0.206
Previous neoplasm ^c^	14 (4.9)	6 (6.4)	8 (4.2)	0.719
Chronic neurological disorders	29 (10.2)	14 (14.9)	15 (7.9)	0.067
Autoimmune diseases	29 (10.2)	8 (8.5)	21 (11.0)	0.507
Other chronical diseases	38 (13.4)	12 (12.7)	26 (13.7)	0.831
**Clinical characteristics**				
Previous surgery in last month	11 (3.9)	4 (4.2)	7 (3.7)	0.815
Previous hospitalization last 6 months	21 (7.4)	7 (7.4)	14 (7.4)	0.981
Pre-ICU hospitalization, days	6 (± 8.0)	4.7 (± 5.5)	6.6 (± 9.0)	0.072
Total days on mechanical ventilation	26.9 (± 19.9)	32.0 (± 18.6)	24.3 (± 20.0)	**0.002**
ICU length of stay, days	29.3 (± 19.8)	33.4 (± 18.4)	27.3 (± 20.2)	**0.015**
Renal placement therapy, no. (%)	27 (9.5)	11 (11.7)	16 (8.4)	0.835
Barotrauma, no. (%)	19 (6.7)	4 (4.3)	15 (7.9)	0.250
Pronation ^d^	109 (38.4)	32 (34)	77 (40.5)	0.282
Blood transfusion	104 (36.2)	45 (47.8)	59 (31.0)	0.006
Tocilizumab/Sarilumab	56 (19.5)	26 (27.6)	30 (15.8)	**0.018**
Pre-OTI antibiotic therapy ^e^	149 (51.9)	55 (58.5)	94 (49.5)	0.152
**Outcome**				
ICU discharged, patients, no. %	164 (57.8)	50 (53.2)	114 (60.0)	0.276
ICU mortality, patients, no. %	120 (42.2)	44 (46.8)	76 (40.0)	0.276
30-day mortality, no. %	104 (36.6)	35 ((37.2)	69 (36.3)	0.880
60-day mortality, no. %	119 (41.9)	43 (45.7)	76 (40)	0.358

*IQR* Interquartile range, *ICU* Intensive care unit, *BMI* Body mass index, *SOFA score Sequential organ failure assessment and APACHE II score, acute physiologic, and chronic health evaluation at ICU admission. ^a^Obesity is defined as BMI > 30 kg/m^2^; ^b^Stages 3–5 of CKD, chronic kidney disease; ^c^Includes solid neoplasia or hematological malignancy in the last 5 years; ^d^At least one cycle of 12 h; ^e^30 days before orotracheal intubation

As shown in Table 1, renal placement therapy was used in 27 patients (9.5%), 109 patients (38.4%) underwent at least 10 h of pronation prior to the development of the VAP, 56 patients (19.5%) were treated with Tocilizumab or Sarilumab prior OTI, and 149 patients (51.9%) were treated with empirical antibiotic therapy prior OTI. At 30 days from ICU admission, 104 patients (36.6%) died and the overall ICU mortality rate was 42.2%.

### Prevalence and incidence of VAP in ICU COVID-19 patients

During the study period, 103 episodes of VAP were diagnosed in 94 patients (9 patients had more than one episode), and the prevalence was 33% (94/284). The incidence rate of VAP was 13.48 (103 episodes/7638 days on MV) episodes per 1000 days in MV.

The etiology of the VAPs and its incidence are shown on Table 2. The incidence of Gram-negative bacteria was higher than that of Gram-positive bacteria, 12.4 and 1.04 VAPs per 1000 days on MV, respectively. The main etiological agents were *Pseudomonas aeruginosa* (41 episodes, 39.8% of all VAPs) of which 17 (41.4%) were carbapenem resistant, *Klebsiella spp*. (17 episodes, 16.5%) of which 3 (17.6%) were carbapenem resistant, *Staphilococcus aureus* (8 episodes, 7.7%) of which 4 (57%) were methicillin-resistant, *Acinetobacter baumanii* (8 episodes, 7.7%) of which all of them were carbapenem resistant, *Stenotrophomonas maltophilia* (8 episodes, 7.7%), *Escherichia coli* (6 episodes, 5.8%), *Serratia marcescens* (4 episodes, 4.8%), and *Proteus spp*. (4 episodes, 3.8%).

**Table 2:** Number of isolated microorganisms in ventilator-associated pneumonia episodes

Microorganism	**No. of VAPs**	**In OTI**	**In tracheostomy**
**Gram-positive**	**8**	**7**	**1**
Incidence (VAPs/1000 MV days)	1.04	1.7	0.3
Methicillin-sensitive *Staphylococcus aureus*	4	3	1
Methicillin-resistant *Staphylococcus aureus*	4	4	0
**Gram-negative**	**95**	**62**	**33**
Incidence (VAPs/1000 MV days)	12.4	14.8	9.6
*Pseudomonas aeruginosa (Carba-R)*	41 (17)	24 (5)	17 (12)
*Klebsiella species (Carba-R)*	17 (3)	12 (0)	5 (3)
*K. pneumoniae ****(****Carba-R)*	10 (2)	7 (0)	3 (2)
*K. aerogenes (Carba-R)*	6 (1)	4 (0)	2 (1)
*K. oxytoca*	1	1	0
*Stenotrophomonas maltophilia*	8	6	2
*Acinetobacter spp.*	8	7	1
*Escherichia coli*	6	4	2
*Serratia marcescens*	5	3	2
*Proteus spp.*	4	0	4
*Enterobacter cloacae*	3	3	0
Others	3	3	0
**Total mechanical ventilation time, days**	7638 days	4192 days	3446 days
Total VAP episodes	103	69	34
Incidence of VAP per 1000 MV days	13.48	16.46	9.86

*VAP* Ventilator-associated pneumonia, *Carba-R* Carbapenem-resistant, *ICU* Intensive care unit, *OTI* Orotracheal intubation, *MV* Mechanical ventilation

The accumulative total MV time in orotracheal tube was 4192 days and in tracheostomy were 3446 days. Patients during the mechanical ventilation in OTI had a higher overall incidence than those in tracheostomy, 16.46 (69/4192) and 9.86 (34/3446) episodes per 1000 days on MV, respectively.

**Fig. 3:**
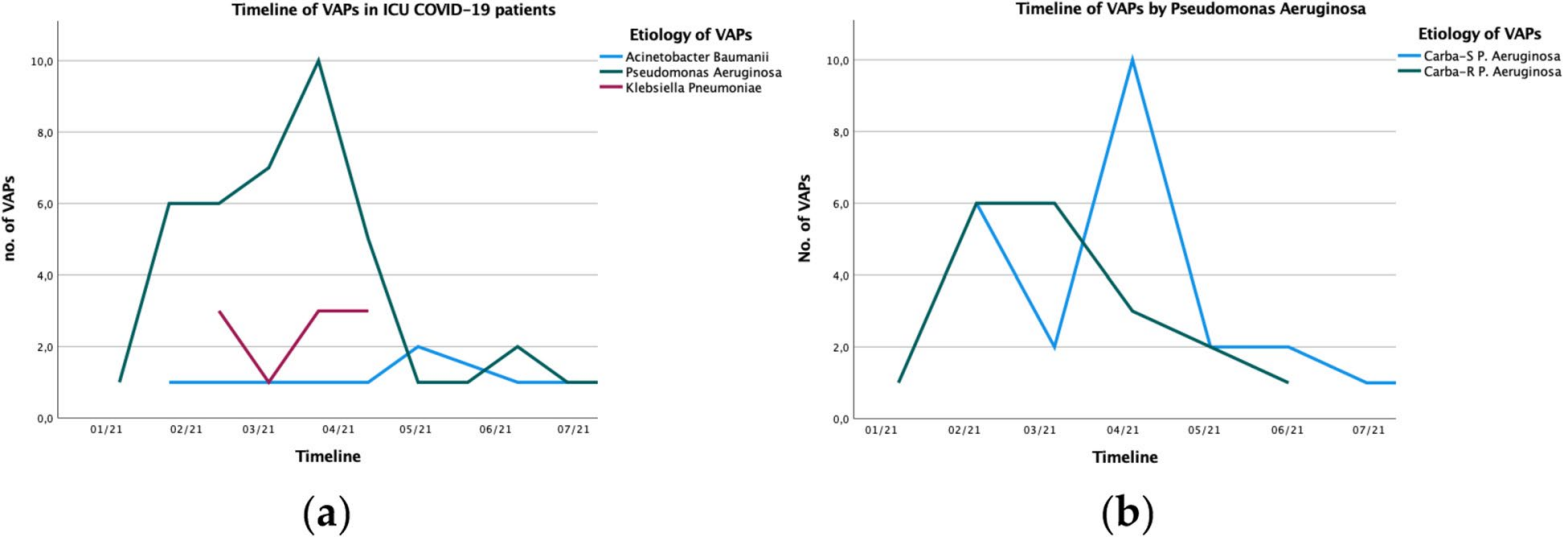
Timeline of aetiology of VAPs. VAP, ventilator-associated pneumonia; ICU, intensive care unit; Carba-R, carbapenem-resistant; Carba-S, carbapenem-susceptible

*Pseudomonas aeruginosa and Klebsiella spp*. had an incidence respectively of 5.36 and 2.2 episodes per 1000 ventilator days. The timeline of the main bacterial agent, as *Acinetobacter baumanii*, *Pseudomonas aeruginosa*, and *Klebsiella spp*, during the period of study are shown in Fig. 3a and b shows of *Pseudomonas aeruginosa* carbapenem susceptible vs resistant.

### Risk factors for VAP in ICU COVID-19 patients

**Fig. 4:**
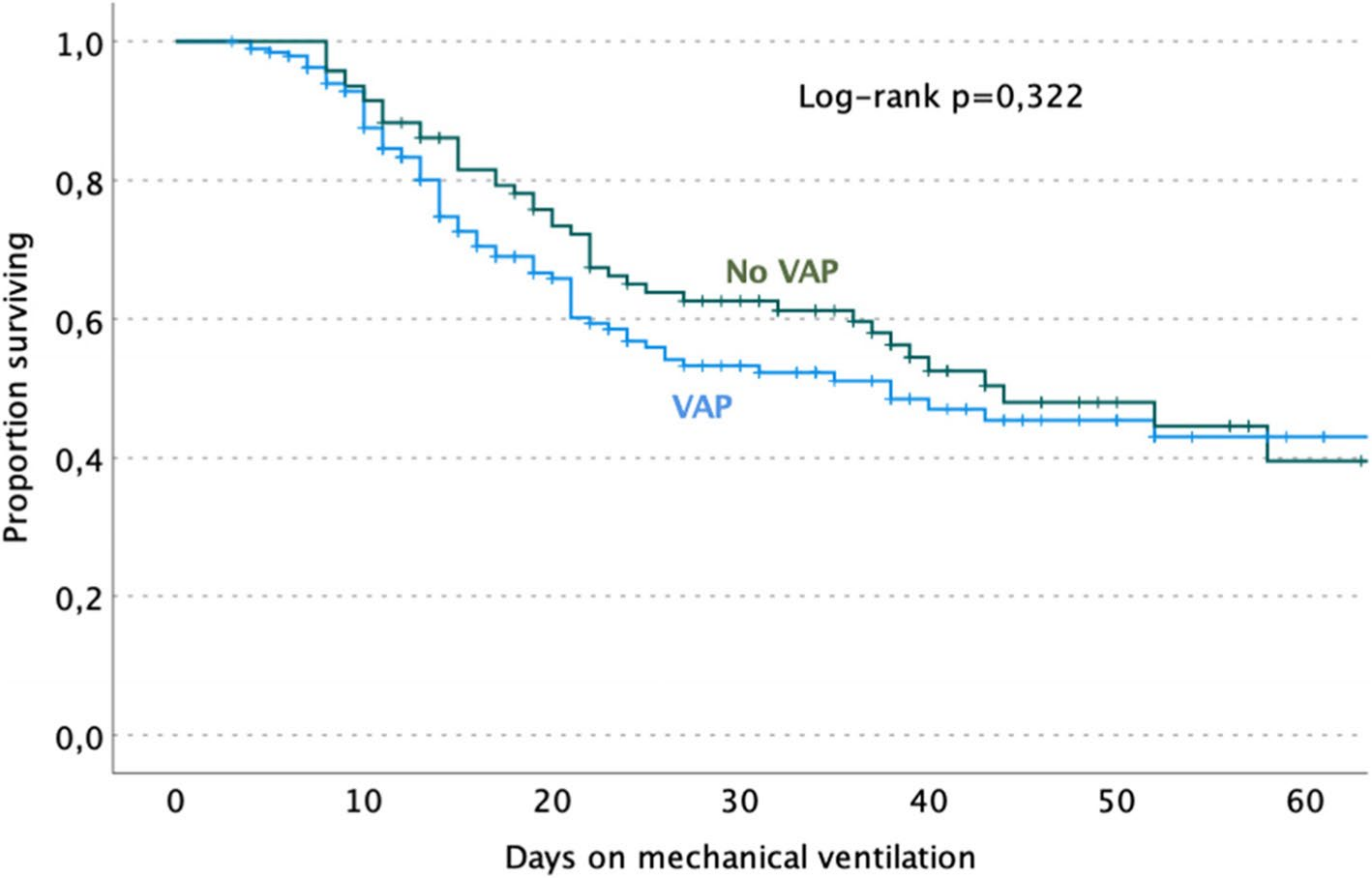
Probability of survival at 60 days of follow-up in patients with the first ventilator-associated pneumonia and without it

At univariate analysis, determinant factors significantly associated with VAP were SOFA score ≥ 4 at ICU admission with a twice higher risk (OR 2.19, 95% CI 1.26–3.81, *p* = 0.005), female with a lower risk than male (OR 0.58, 95% CI 0.34–0.99, *p* = 0.045); blood transfusions prior to VAP with a twofold higher risk (OR 2.04, 95% CI 1.23–3.39, *p* = 0.006) (Table 3). Administration of Tocilizumab or Sarilumab was associated with a twofold higher risk (OR 2.03, 95% CI 1.12–3.70, *p* = 0.018) and ICU length of stay > 28 days with over twice higher risk (OR 2.29, 95% CI 1.38–3.79). Comorbidities, corticosteroid therapy, pronation, antibiotic therapy prior-OTI, and P/F ratio at ICU admission were determinant factors not significantly associated with the development of VAP. Moreover, VAP episodes did not significantly increase the mortality at 28 days from ICU admission and the overall ICU mortality rate (Fig. 4).

**Table 3:** Unadjusted and adjusted risk factors for developing at least one VAP in ICU COVID-19 patients

Variable	Total ICU patients	VAP group	Univariate	Multivariate		***p***
			*OR (95% CI)*	*p*	*aOR (95% CI)*	
	284	94 (33)				
Age > 70 years old	111	37 (33.3)	1.02 (0.61–1.69)	0.946		
Female (vs male), ***n*** (%)	203	60 (29.6)	0.58 (0.34–0.99)	**0.045**	0.69 (0.39–1.22)	0.205
SOFA score ≥ 4	182	71 (39)	2.19 (1.26–3.81)	**0.005**	2.11 (1.20–3.71)	**0.009**
APACHE II score ≥ 10	182	67 (36.8)	1.62 (0.95–2.76)	0.076	1.05 (0.55–2.00)	0.883
Comorbidities, ***n*** (%)						
Arterial hypertension	156	51 (32.7)	0.96 (0.58–1.58)	0.872		
Cardiovascular diseases	66	28 (42.4)	1.69 (0.96–2.99)	0.066	1.56 (0.85–2.83)	0.148
Diabetes	50	17 (34)	1.05 (0.55–2.00)	0.881		
Obesity ^a^	100	40 (40)	1.60 (0.96–2.67)	0.068	1.55 (0.91–2.65)	0.106
Chronic lung disease	38	16 (42.1)	1.56 (0.78–3.15)	0.205		
Chronic renal disease ^b^	13	5 (38.5)	1.27 (0.40–4.02)	0.674		
Renal placement therapy	27	11 (40.7)	1.44 (0.64–3.24)	0.375		
Previous neoplasm ^c^	15	6 (40)	1.37 (0.47–3.97)	0.559		
Chronic neurological disorders	29	14 (48.3)	2.04 (0.94–4.43)	0.067		
Autoimmune diseases	29	8 (27.6)	0.75 (0.32–1.76)	0.506		
Other chronical diseases	38	12 (31.6)	0.92 (0.44–1.92)	0.830		
Clinical characteristics						
Corticosteroid therapy	201	69 (34.3)	1.21 (0.69–2.10)	0.493		
Pronation ^d^	109	32 (29.4)	0.76 (0.45–1.27)	0.290		
Barotrauma	19	4 (21.1)	0.52 (0.17–1.60)	0.248		
Blood transfusion	104	45 (43.3)	2.04 (1.23–3.39)	**0.006**	2.13 (1.26–3.59)	**0.005**
Tocilizumab/Sarilumab	56	26 (46.4)	2.03 (1.12–3.70)	**0.018**	2.08 (1.12–3.84)	**0.020**
Pre-OTI antibiotic therapy	149	55 (36.9)	1.44 (0.87–2.37)	0.151		
PaO_2_/FiO_2_ ≤ 100 mmHg	48	16 (33.3)	1.01 (0.52–1.95)	0.970		
ICU length of stay > 28 days	113	50 (44.2)	2.29 (1.38–3.79)	**0.001**	1.89 (1.10–3.24)	**0.020**
30-day ICU mortality	104	35 (33.7)	1.04 (0.62–1.73)	0.880		
Overall ICU mortality	120	44 (36.7)	1.32 (0.80–2.17)	0.274		

*IQR* Interquartile range, *ICU* Intensive care unit, *BMI* Body mass index, *SOFA score* Sequential organ failure assessment and *APACHE II score*, Acute physiologic and chronic health evaluation at ICU admission. ^a^Obesity is defined as BMI > 30 kg/m^2^; ^b^Stages 3–5 of CKD, chronic kidney disease; ^c^Includes solid neoplasia or hematological malignancy in the last 5 years; ^d^At least one cycle of 12 h

The multiple regression analysis shows that ≥ 4 SOFA score (OR 2.11, 95% CI 1.20–3.71, *p* = 0.009), blood transfusion prior to VAP (OR 2.13, 95% CI 1.26–3.59, *p* = 0.005), treatment with Tocilizumab or Sarilumab (OR 2.08, 95% CI 1.12–3.84, *p* = 0.02), and > 28-day ICU LOS (OR 1.89, 95% CI 1.10–3.24) were all independent factors related to a twice higher risk of developing VAP.

## Discussion

This study shows a 33% crude prevalence rate of VAP and an overall incidence density of 13.48 episodes per 1000 days of MV, higher than the incidence in non-COVID patients from the report of the Italian project GiViTi of 2019 (with an incidence of 9.8 events per 1000 ventilator days) [7] and of the European intensive care units (report of ECDC) in 2019 (9.5 episodes per 1000 ventilator days) [6]. However, the incidence of VAP is quite similar compared to studies including only ARDS patients as reported by Guerin et al. for PROSEVA trial in 2016 (between 11.8 and 15.4 VAP per 1000 ventilator days) [16].

VAP did not significantly increase the ICU mortality rate in our cohort, consistently with previous reports in COVID-19 [12], and non-COVID-19 patients, even with severe ARDS [7]. Improvement have occurred in the past few years in the management of ARDS patients in the ICU. Lung-protective ventilation strategies have been widely used in ARDS patients the past few decades and especially in the last 2 years of pandemic with COVID-19 ARDS patients [17, 18]. These strategies together with prompt antibiotic treatment may contribute to explaining this finding, as also some evidence indicates that cyclic stretch of pulmonary epithelial cells may represent an important pathogenic mechanism of VAP, promoting bacterial growth [19].

The sequential organ failure assessment (SOFA) score at ICU admission ≥ 4 and ICU length of stay > 28 days were independently associated with higher risk of VAP, although ICU length of stay could be as much a cause as a consequence. SOFA score has been developed to assess the acute morbidity of critical illness and is widely used in predicting mortality and ICU length of stay in septic patients [20], as in some studies, it has been shown that it can also be a predictor for ventilator-associated pneumonia [21].

Ventilated patients in OTI had a 1.7-fold higher incidence of VAP than those in tracheostomy, 16.46 and 9.8 episodes per 1000 days on MV, respectively. Indeed, tracheostomy has many beneficial effects in MV patients, facilitating the weaning process, reducing the need for sedative and analgesics, reducing dead space and airway resistance, and finally, helping to maintain easier oral hygiene [22].

In our cohort, the most frequently isolated microorganisms were *Pseudomonas aeruginosa* (39.8%), *Klebsiella spp*. (16.5%), *Staphylococcus aureus* (7.7%), *Stenotrophomonas maltophylia* (7.7%), *Acinetobacter baumanii* (7.7%), and *Escherichia coli* (5.8%). Although some of the isolated microorganisms of our study are overlapping with those of the 2019 ECDC report, others such as *P. aeruginosa*, *Klebsiella spp*, and *A. baumanii* have higher incidence. This is likely to be due to the SARS-CoV-2-related upper respiratory tract microbiome dysbiosis reported in some studies, correlated to a profoundly altered microbiome profile through different mechanisms, as known for other respiratory viruses, including alteration of the respiratory epithelium, promotion of adhesion of respiratory pathogens, and increase of local inflammation [23, 24].

Carbapenem resistance resulted in all *Acinetobacter spp*. and in 41.4% of *P. aeruginosa* isolates with a 56% and 60% increase over the ECDC report in 2019 (63.9% and 25.9%, respectively). Instead, carbapenem resistance in *Klebsiella spp*. isolates was 17.6%, which is slightly higher to that reported by ECDC in 2019 (15.2%). Half of *S. aureus* were methicillin-resistant (MRSA) with a 113% increase over ECDC report in 2019 (23.5%). Widespread and inappropriate prescription of broad-spectrum antibiotics as prophylaxis for COVID-19 (usually beta-lactams, macrolides, and fluoroquinolones) has been observed during the pandemic, as discussed in the 32nd European Congress of Clinical Microbiology & Infectious Diseases (ECCMID), presented on April 23–26, 2022 [25], showing high rates of antimicrobial resistance in COVID-19 patients. As expected, also in our study, a high percentage of our patients (52.4%) used broad-spectrum antibiotics prior to ICU admission and often started before admission to hospital. The overuse of empirical antibiotics before intubation could have led to an unfavorable development of multidrug resistant bacteria, as a high proportion of carbapenem resistance and MRSA microorganisms.

Late-onset VAP were more likely to be caused by multidrug-resistant (MDR) pathogens (Table 2, Supplemental Material).

Demographics (age, sex), comorbidities, renal placement therapy, PaO_2_/FiO_2_ at ICU admission, and empirical antibiotic therapy prior OTI were not associated with a higher risk of VAP, consistently with the data reported by Blonz et al. [11]. Barotrauma, as pneumothorax or pneumomediastinum, in our ICU COVID-19 patients had a relatively low incidence [26], and it was not associated with a higher risk of VAP. Moreover, blood transfusion during the ICU stay had an independent higher risk of developing VAPs, as reported in a multicenter study including 284 intensive care units in the USA in non-COVID-19 population, by Shorr et al. [27]. An experimental hypothesis is that blood transfusion promotes the release of cytokines, which enhances various cytokine cascades producing pulmonary inflammation, as well as bronchoalveolar activation of coagulation [28].

The patient’s body position is an important aspect in ICU. A semi-recumbent position (i.e., elevation of the head of bed to 30–45°) has been widely used in our ICU as a strategy for patients undergoing MV as a recommendable measure in several clinical practice guidelines for preventing VAP [29]. This position can help reduce gastroesophageal reflux and avoid the entry of gastric contents and contaminated oropharyngeal secretions into the lower airway [29]. An early application of prone position seems to have a survival benefit in patients with moderate-severe ARDS, promoting the distribution of the gas–tissue ratios along the dependent–nondependent axis and a more homogeneous distribution of lung stress and strain [30]. Indeed, during the pandemic prone position has been a common strategy in COVID-19 patients with refractory hypoxemia [31]. Although the prone position appears not to be associated with a higher risk of VAP, as reported as well by Ayzac et al. in the PROSEVA trial in 2016 and in a recent review by Pozuelo‑Carrascosa et al. in 2022, in intubated patients with severe ARDS, and our results are consistent with them [16, 29].

Interleukin-6 acts as a fundamental cytokine in organizing T cell responses to infections and, eventually, may protect against superinfection. Tocilizumab or Sarilumab are anti IL-6 receptor monoclonal antibodies, approved for the treatment of multiple inflammatory diseases, and have been considered for off-label use in the treatment of COVID-19 [32, 33]. We found that Tocilizumab/Sarilumab had a significant association with a twofold higher risk of VAP at both univariate and multivariate analysis, consistently with data reported by Martinez et al. [34].

This study has a major limitation. This is an observational study conducted in a single center with a 6-month observation period on a limited population of approximately 300 MV COVID-19 patients; however, our prevalence and incidence rate are in line with literature data, and it represent a first step to formulate hypothesis to design of larger clinical studies.

## Conclusions

Our study shows an incidence of VAP in COVID-19 patients higher than that reported in non-COVID-19 patients [6], but in line with the data reported in ARDS patients in pre-COVID era [16]. Patients with tracheostomy had a reduced incidence of VAPs, and a correct timing of tracheostomy in MV patients is a key strategy to reduce VAP. Conversely, interleukin-6 inhibitors and blood transfusion in our COVID-19 patients have been all recognized as independent risk factors for developing VAP. Prone position did not increase this risk.

We reported a high incidence of *carbapenem resistance Pseudomonas aeruginosa* and *MRSA*, likely related to an increased use of empirical antibiotics. Since management practices for COVID-19 patients in the ICU are constantly evolving, the widespread use of empirical antibiotics in these patients and optimal infection control measures by implementation of antimicrobial stewardship programs should be all taken into consideration, to avoid the development of multidrug-resistant bacteria.

Finally, VAP did not increase the ICU case mortality rate.

## Supplementary Information

**Additional file 1: Table 1.** Comparison of demographic and clinical characteristics of patients by early and late VAP. **Table 2.** Number of isolated microorganisms in ventilator-associated pneumonia episodes by early and late VAP.

## Data Availability

The data presented in this study are available on request from the corresponding author. The data are not publicly available because of patient privacy and data protection regulations.

## Abbreviations

COVID-19: Coronavirus 2019
ICU: Intensive care unit
MV: Invasive mechanical ventilation
NIV: Non-invasive ventilation
VAP: Ventilator-associated pneumonia
OTI: Orotracheal intubation
ARDS: Acute respiratory distress syndrome
IQR: Interquartile range
BMI: Body mass index
SOFA score: Sequential organ failure assessment
APACHE II score: Acute physiologic and chronic health evaluation
PaO2/FiO2 ratio: The ratio of arterial partial pressure (PaO2) to fractional inspired oxygen (FiO2)
CKD: Chronic kidney disease
Carba-R: Carbapenem-resistant
Carba-S: Carbapenem-susceptible

## References

[1] WHO Coronavirus Disease (COVID-19) Dashboard | WHO coronavirus disease (COVID-19) Dashboard Available online: https://covid19.who.int/ (Accessed on 27 May 2022).

[2] Privitera D, Angaroni L, Capsoni N, Forni E, Pierotti F, Vincenti F, Bellone A (2020). Flowchart for non-invasive ventilation support in COVID-19 patients from a Northern Italy Emergency Department. Intern Emerg Med.

[3] Mehta C, Mehta Y (2017). Percutaneous tracheostomy. Ann Card Anaesth.

[4] Koulenti D, Tsigou E, Rello J (2017). Nosocomial pneumonia in 27 ICUs in Europe: perspectives from the EU-VAP/CAP study. Eur J Clin Microbiol Infect Dis.

[5] Torres A, Niederman MS, Chastre J, Ewig S, Fernandez-Vandellos P, Hanberger H, Kollef M, Li Bassi G, Luna CM, Martin-Loeches I (2017). International ERS/ESICM/ESCMID/ALAT guidelines for the management of hospital-acquired pneumonia and ventilator-associated pneumonia. Eur Respir J.

[6] European Centre for Disease Prevention and Control. Healthcare-associated infections acquired in intensive care units. In: ECDC. Annual epidemiological report for 2017. Stockholm: ECDC; 2019. https://www.ecdc.europa.eu/en/publications-data/healthcare-associated-infections-intensive-care-units-annual-epidemiological-1. Accessed 27 May 2022.

[7] GiViTi, G.I. per la V. degli I. in T.I. GiViTi, Gruppo Italiano per La Valutazione Degli Interventi in Terapia Intensiva. Annual Epidemiological Report for 2018 Progetto PROSAFE - Petalo INFEZIONI 2018; Bergamo; 2019. https://giviti.marionegri.it/attachments/Projects/Infection/ReportPetaloInfezioni_2019_IT_TIPolivalenti.pdf. Accessed on 27 May 2022.

[8] Forel J-M, Voillet F, Pulina D, Gacouin A, Perrin G, Barrau K, Jaber S, Arnal J-M, Fathallah M, Auquier P (2012). Ventilator-associated pneumonia and ICU mortality in severe ARDS patients ventilated according to a lung-protective strategy. Crit Care.

[9] Grasselli G, Scaravilli V, Mangioni D, Scudeller L, Alagna L, Bartoletti M, Bellani G, Biagioni E, Bonfanti P, Bottino N (2021). Hospital-acquired infections in critically ill patients with COVID-19. Chest.

[10] Giacobbe DR, Battaglini D, Enrile EM, Dentone C, Vena A, Robba C, Ball L, Bartoletti M, Coloretti I, di Bella S (2021). Incidence and prognosis of ventilator-associated pneumonia in critically ill patients with COVID-19: a multicenter study. J Clin Med.

[11] Blonz G, Kouatchet A, Chudeau N, Pontis E, Lorber J, Lemeur A, Planche L, Lascarrou J-B, Colin G (2021). Epidemiology and microbiology of ventilator-associated pneumonia in COVID-19 patients: a multicenter retrospective study in 188 patients in an un-inundated French region. Crit Care.

[12] Ippolito M, Misseri G, Catalisano G, Marino C, Ingoglia G, Alessi M, Consiglio E, Gregoretti C, Giarratano A, Cortegiani A (2021). Ventilator-associated pneumonia in patients with COVID-19: a systematic review and meta-analysis. Antibiotics.

[13] Kalil AC, Metersky ML, Klompas M, Muscedere J, Sweeney DA, Palmer LB, Napolitano LM, O’Grady NP, Bartlett JG, Carratalà J (2016). Management of adults with hospital-acquired and ventilator-associated pneumonia: 2016 clinical practice guidelines by the infectious diseases Society of America and the American Thoracic Society. Clin Infect Dis.

[14] Singer P, Blaser AR, Berger MM, Alhazzani W, Calder PC, Casaer MP, Hiesmayr M, Mayer K, Montejo JC, Pichard C (2019). ESPEN guideline on clinical nutrition in the intensive care unit. Clin Nutr.

[15] Tetaj N, Maritti M, Stazi G, Marini MC, Centanni D, Garotto G, Caravella I, Dantimi C, Fusetti M, Santagata C (2021). Outcomes and timing of bedside percutaneous tracheostomy of COVID-19 patients over a year in the intensive care unit. Journal of Clinical Medicine.

[16] Ayzac L, Girard R, Baboi L, Beuret P, Rabilloud M, Richard JC, Guérin C (2016). Ventilator-associated pneumonia in ARDS patients: the impact of prone positioning. A Secondary Analysis of the PROSEVA Trial. Intensive Care Medicine.

[17] Marini JJ, Gattinoni L (2020). Management of COVID-19 respiratory distress. JAMA.

[18] Brochard L, Slutsky A, Pesenti A (2017). Mechanical ventilation to minimize progression of lung injury in acute respiratory failure. Am J Respir Crit Care Med.

[19] Pugin J, Dunn-Siegrist I, Dufour J, Tissières P, Charles P-E, Comte R (2008). Cyclic stretch of human lung cells induces an acidification and promotes bacterial growth. Am J Respir Cell Mol Biol.

[20] Lambden S, Laterre PF, Levy MM, Francois B (2019). The SOFA score—development, utility and challenges of accurate assessment in clinical trials. Crit Care.

[21] Boeck L, Eggimann P, Smyrnios N, Pargger H, Thakkar N, Siegemund M, Morgenthaler NG, Rakic J, Tamm M, Stolz D (2012). The sequential organ failure assessment score and copeptin for predicting survival in ventilator-associated pneumonia. J Crit Care.

[22] Trouillet JL, Collange O, Belafia F, Blot F, Capellier G, Cesareo E, Constantin JM, Demoule A, Diehl JL, Guinot PG (2018). Tracheotomy in the intensive care unit: guidelines from a French expert panel: the French Intensive Care Society and the French Society of Anaesthesia and Intensive Care Medicine. Anaesth Crit Care Pain Med.

[23] Soffritti I, D’Accolti M, Fabbri C, Passaro A, Manfredini R, Zuliani G, Libanore M, Franchi M, Contini C, Caselli E (2021). Oral microbiome dysbiosis is associated with symptoms severity and local immune/inflammatory response in COVID-19 patients: a cross-sectional study. Front Microbiol.

[24] Shilts MH, Rosas-Salazar C, Strickland BA, Kimura KS, Asad M, Sehanobish E, Freeman MH, Wessinger BC, Gupta V, Brown HM (2022). Severe COVID-19 is associated with an altered upper respiratory tract microbiome. Front Cell Infect Microbiol.

[25] 32nd European Congress of Clinical Microbiology & Infectious Diseases (ECCMID) April 23–26, 2022.; European Society of Clinical Microbiology and Infectious Diseases: Lisbon, Portugal, 2022. https://www.eccmid.org (Accessed on 27 May 2022).

[26] Tetaj N, Garotto G, Albarello F, Mastrobattista A, Maritti M, Stazi GV, Marini MC, Caravella I, Macchione M, de Angelis G (2021). Incidence of pneumothorax and pneumomediastinum in 497 COVID-19 patients with moderate–severe ards over a year of the pandemic: an observational study in an Italian third level COVID-19 hospital. J Clin Med.

[27] Shorr AF, Duh M-S, Kelly KM, Kollef MH (2004). Red blood cell transfusion and ventilator-associated pneumonia: a potential link?. Crit Care Med.

[28] Tuinman PR, Vlaar AP, Cornet AD, Hofstra JJ, Levi M, Meijers JCM, Beishuizen A, Schultz MJ, Groeneveld AJ, Juffermans NP (2011). Blood transfusion during cardiac surgery is associated with inflammation and coagulation in the lung: a case control study. Crit Care.

[29] Pozuelo-Carrascosa DP, Cobo-Cuenca AI, Carmona-Torres JM, Laredo-Aguilera JA, Santacruz-Salas E, Fernandez-Rodriguez R (2022). Body position for preventing ventilator-associated pneumonia for critically ill patients: a systematic review and network meta-analysis. J Intensive Care.

[30] Gattinoni L, Taccone P, Carlesso E, Marini JJ (2013). Prone position in acute respiratory distress syndrome. Rationale, Indications, and Limits. Am J Respir Crit Care Med.

[31] Langer T, Brioni M, Guzzardella A, Carlesso E, Cabrini L, Castelli G, Dalla Corte F, de Robertis E, Favarato M, Forastieri A (2021). Prone position in intubated, mechanically ventilated patients with COVID-19: a multi-centric study of more than 1000 patients. Critical Care.

[32] Salama C, Han J, Yau L, Reiss WG, Kramer B, Neidhart JD, Criner GJ, Kaplan-Lewis E, Baden R, Pandit L (2021). Tocilizumab in patients hospitalized with COVID-19 pneumonia. N Engl J Med.

[33] Khiali S, Rezagholizadeh A, Entezari-Maleki T (2021). A comprehensive review on sarilumab in COVID-19. Expert Opin Biol Ther.

[34] Martínez-Martínez M, Plata-Menchaca EP, Nuvials FX, Roca O, Ferrer R (2021). Risk factors and outcomes of ventilator-associated pneumonia in COVID-19 patients: a propensity score matched analysis. Crit Care.

