# Supplementary material for: Epidemiology of ventilator-associated pneumonia in ICU COVID-19 patients: an alarming high rate of multidrug-resistant bacteria
Source: J Anesth Analg Crit Care. 2022 Aug 19;2:36. doi: 10.1186/s44158-022-00065-4 (PMC9389502; doi:10.1186/s44158-022-00065-4)
Supplement: Supplementary file 1 — Additional file 1: Table 1. Comparison of demographic and clinical characteristics of patients by early and late VAP. Table 2. Number of isolated microorganisms in ventilator-associated pneumonia episodes by early and late VAP. [file 44158_2022_65_MOESM1_ESM.docx]

SUPPLEMENT MATERIAL

Epidemiology of Ventilator-Associated Pneumonia in ICU COVID-19 Patients: An alarming high rate of multidrug resistant bacteria

Nardi Tetaj ^1^, Alessandro Capone ^2^, Giulia Valeria Stazi ^1^, Maria Cristina Marini ^1^, Gabriele Garotto ^1^, Donatella Busso ^1^, Silvana Scarcia ^1^, Ilaria Caravella ^1^, Manuela Macchione ^1^, Giada De Angelis ^1^, Rachele Di Lorenzo ^1^, Alessandro Carucci ^1^, Maria Vittoria Antonica ^1^, Ilaria Gaviano ^1^, Carlo Inversi ^1^, Elisabetta Agostini ^1^, Flaminia Canichella ^1^, Giorgia Taloni ^1^, Francesca Evangelista ^1^, Ilaria Onnis ^1^, Giulia Mogavero ^1^, Maria Elena Lamanna ^1^, Dorotea Rubino ^1^, Mattia Di Frischia ^1^, Candido Porcelli ^1^, Elena Cesi ^1^, Andrea Antinori ^2^, Fabrizio Palmieri ^2^, Gianpiero D’Offizi ^2^, Fabrizio Taglietti ^2^, Carla Nisii ^2^, Maria Adriana Cataldo ^2^, Stefania Ianniello ^2^, Paolo Campioni ^2^, Francesco Vaia ^3^, Emanuele Nicastri ^2^, Enrico Girardi ^4^, Luisa Marchioni ^1^ and ICU COVID-19 Study Group †

* Correspondence: nardi.tetaj@inmi.it, UOC Intensive and Sub-Intensive Care, National

^1^ Institute for Infectious Diseases IRCCS, Lazzaro Spallanzani, Rome, Italy.

Full list of author information is available at the end of the article

Definitions

Early VAP: is defined when a ventilator-associated pneumonia (VAP) occurs within the first 4 days of mechanical ventilation (MV).

Late VAP: is defined when a VAP occurs after 4 days from MV.

**Early versus Late VAP**

Twenty-one patients (22%) had early-onset VAP (within the first 4 days of MV) and 73 of them (78%) had late-onset VAP (> 4 days MV). No significant difference were observed between early and late VAP in the comparison of patient demographics, clinical features, Table 1.

As shown in the Table 2, Late-onset VAP were more likely to be caused by multidrug-resistant (MDR) pathogens, such as *Pseudomonas aeruginosa Carba-R, Klebsiella species Carba-R, and Acinetobacter spp.*

**Table 1.** Comparison of demographic and clinical characteristics of patients by early and late VAP.

| Characteristics | Patients with  early VAP | Patients with  late VAP | p-value ^a^ |
| --- | --- | --- | --- |
|  | 21 | 73 |  |
| Age, median (IQR) | 65 (54-74) | 68 (58-73) | 0.429 |
| Male, n (%) | 14 (66.7) | 46 (63) | 0.762 |
| Female, n (%) | 7 (33.3) | 27 (37) | 0.762 |
| BMI, kg/m2, median (IQR) | 26.2 (24.5-33.2) | 28.7 (25.5-32.1) | 0.617 |
| SOFA score, median* (IQR) | 4 (3-7) | 6 (4-8) | 0.399 |
| APACHE II score, median* (IQR) | 14 (9-18) | 13 (9-20) | 0.717 |
| Number of comorbidities, n (%) |  |  |  |
| 0 | 4 (19) | 10 (13.7) | 0.549 |
| 1 | 7 (33.3) | 16 (21.9) | 0.289 |
| 2 | 5 (23.8) | 21 (28.7) | 0.659 |
| ≥3 | 5 (23.8) | 26 (35.6) | 0.316 |
| Outcome |  |  |  |
| ICU discharged, patients, no. % | 9 (42.8) | 41 (56.2) | 0.286 |
| ICU mortality, patients, no. % | 12 (57.1) | 32 (43.8) | 0.286 |
| 30-day mortality, no. % | 11 ((52.3) | 24 (32.8) | 0.105 |
| 60-day mortality, no. % | 11 ((52.3) | 32 (43.8) | 0.494 |

IQR, interquartile range**;** ICU, intensive care unit; BMI, body mass index; *SOFA score, sequential organ failure assessment and APACHE II score, acute physiologic and chronic health evaluation at ICU admission.

**Table 2.** ﻿Number of isolated microorganisms in ventilator-associated pneumonia episodes by early and late VAP.

|  | All VAPs | Early VAP | Late VAP |
| --- | --- | --- | --- |
| Gram-positive | **8** | **3** | **5** |
| Methicillin-sensitive *Staphylococcus aureus*  Methicillin-resistant *Staphylococcus aureus* | 4  4 | 2  1 | 2  3 |
| Gram-negative | **95** | **24** | **71** |
| *Pseudomonas aeruginosa (Carba-R)* | 41 (17) | 8 (3) | 33 (14) |
| *Klebsiella species (Carba-R)*  *K. pneumoniae (Carba-R)*  *K. aerogenes (Carba-R)*  *K. oxytoca* | 17 (3)  10 (2)  6 (1)  1 | 6 (1)  3 (1)  3 (0)  0 | 11 (2)  7 (1)  3 (1)  1 |
| *Stenotrophomonas maltophilia* | 8 | 0 | 8 |
| *Acinetobacter spp.* | 8 | 0 | 8 |
| *Escherichia coli* | 6 | 1 | 5 |
| *Serratia marcescens* | 5 | 2 | 3 |
| *Proteus spp.* | 4 | 0 | 4 |
| *Enterobacter cloacae* | 3 | 0 | 3 |
| Others | 3 | 1 | 2 |

VAP, ventilator-associated pneumonia; Carba-R, carbapenem-resistant; ICU, intensive care unit; MV, mechanical ventilation.
